# Disaster after disaster: the outbreak of infectious diseases in Pakistan in the wake of 2022 floods

**DOI:** 10.1097/MS9.0000000000001597

**Published:** 2023-12-08

**Authors:** Marcel Alied, Abdus Salam, Sayed Mansoor Sediqi, Patrick Amanning Kwaah, Linh Tran, Nguyen Tien Huy

**Affiliations:** a Faculty of Pharmacy, University of Aleppo, Aleppo, Syria; b Online Research Club (www.onlineresearchclub.org); c School of Tropical Medicine and Global Health, Nagasaki University, Nagasaki, Japan; d General Surgery department, Khyber Teaching Hospital, Peshawar, Pakistan; e NOOR Eye Training Center, International Asistance Mission, Kabul, Afghanistan; f Department of Internal Medicine, Yale School of Medicine, Yale-Waterbury Internal Medicine Program, Waterbury, Connecticut; g Institute of Fundamental and Applied Sciences, Duy Tan University, Ho Chi Minh City; h Faculty of Natural Sciences, Duy Tan University, Da Nang City, Vietnam

**Keywords:** flooding, pakistan, polio, vector-borne diseases, water-borne diseases

## Abstract

In June 2022, Pakistan witnessed catastrophic floods, affecting millions of people. The ensuing epidemics of cholera, cryptosporidiosis, rotavirus infections, generalized diarrhoea, typhoid and paratyphoid fevers, as well as the frequency of vector-borne diseases including malaria and dengue fever, are studied in this investigation. It also explores the latest outbreak of poliomyelitis and the frequency of respiratory diseases such COVID-19, diphtheria, and tuberculosis, as well as how floods have contributed to skin and eye problems. The report also describes the obstacles governments must overcome in order to manage these health emergencies and offers possible solutions for reducing the effects of ongoing and anticipated epidemics. This flood emphasizes the pressing need for international action and acts as an alarming indicator of the significant impact of climate change. It emphasizes how crucial it is to have effective flood response and preparation strategies in developing nations that are vulnerable to natural disasters.

## Introduction

With a population of 241.5 million, Pakistan is the most populated developing nation in south Asia^1^. Pakistan is experiencing significant economic hardship due to low reserves, currency devaluation, high inflation, and sluggish GDP growth. These issues are made worse by regulatory restraints, flood-related effects, import restrictions, high borrowing and gasoline prices, poor confidence, and protracted political and policy instability^2^. The 2018 health indicators for Pakistan show a complex picture. The incidence of stunting is significant (37.6%), and maternal mortality ratio is high (169 per 100 000 live births), despite improvements in the prevalence of malnutrition (7.1%) and the under-five mortality rate (74 per 1000 live births). The prevalence of diseases is still a problem, particularly TB (269 cases per 100 000 people) and malaria (1.7 cases per 1000 people). Improvements in family planning satisfaction (49%) and vaccination coverage (75.5%) have been made thanks to efforts, but access to comprehensive healthcare services (47.5% coverage) and the number of healthcare workers per 10 000 people still need to be addressed. Important factors to take into account include addressing the overall fertility rate (3.6) and empowering women’s reproductive health decision-making (31.4%)^3^.

At a time when the world is suffering the consequences of climate change, a serious international commitment towards countering what is possibly the greatest threat to mankind is yet to be seen. Heatwaves, droughts, heavy rainfalls, floods, air pollution and a more favourable environment for some diseases vectors have all been witnessed in 2022. Varying precipitation patterns and unpredictable monsoon seasons are yet another repercussion of climate change. Several devastating floods hit South Asian countries in May 2022, shortly before the advent of the monsoon season; however, the worst was yet to come a month later.

By the end of August 2022, one-third of Pakistan was flooded with water and critical health problems. The country witnessed excessive monsoon precipitation in June and consequently the Indus River overflowed. Earlier in March, a heatwave scorched parts of the country resulting in tens of deaths. The record temperatures accelerated the melting of the glaciers that are abundant in the northern parts of the country, releasing glacial melt into rivers basins and thus further fuelling the disastrous floods. According to the National Disaster Management Authority, the floods claimed the lives of 1693 people as of 30 September since 14 June 2022 and inflicted 12 865 injuries^4^. The disaster affected 33 million people living in 84 districts, predominantly in Sindh, Balochistan, Punjab and Khyber Pakhtunkhwa provinces. It is estimated that ~8 million people have been displaced, of which nearly 600 000 sought refuge in camps^5^. Over 2 million houses have been damaged, of which almost 770 000 were entirely ruined. The floods caused heavy damage to the infrastructure affecting roads and bridges and distributing the railway network, which hampered consequently rescue efforts. The havoc wreaked by the floods affected the livestock as well. Over a million head of cattle were killed, with the greatest losses occurring in Balochistan province^4^, where a large proportion of households depend on animal husbandry for their livelihood. As for agricultural land, more than 1.2 million hectares of land was damaged solely in Sindh as of 9 September 2022^6^. Education was interrupted for millions of children and gender-based violence increased.

The poverty-ridden country is devastated by this formidable threat. Although Pakistan’s contribution to climate change is minimal, as put by its climate change minister, yet it is suffering the result of the international indifference towards the climate change threat. However, the poor disaster preparedness and the absence of flood-resilient urban planning is undeniable sin of the government. Nonetheless, the government responded promptly in the wake of the menace, and it is leading the humanitarian action alongside the local communities and non-governmental organizations. On 25 August 2022, they declared a state of emergency. Thousands of medical camps were established in Sindh and thousands of healthcare workers were deployed to the province to provide medical relief to the survivors^7^. The prime minister noted that the damage inflicted by the flood is estimated at 10 billion dollars naming it the worst in the country’s history. The secretary-general of the United Nations pointed out on the other hand that 30 billion dollars are needed for the country to recover from the disaster.

## Public health crisis

Floods cause the destruction of healthcare facilities, inaccessibility to available facilities, shortage in medical supplies and loss of healthcare workers. It has been estimated that over 1460 healthcare facilities have been damaged in Pakistan since June^6^. Almost 24 000 schools have been damaged and over 5000 schools were being used as relief camps^5^. Inequality in the provided healthcare services between urban and rural areas is a long-entrenched issue in the country. This disparity has potentially hindered the healthcare efforts in small towns and villages that turned to be the most affected by the current floods. A study in 2012 concluded that the most socioeconomically deprived regions of Pakistan are the most vulnerable to the adverse impact of climate change, namely Balochistan, Low-Intensity Punjab, and Cotton-Wheat Sindh, thus necessitating socioeconomic betterment in these regions^8^.

Floods have devastating impact on human health. The immediate health risks include drowning, injuries, burns, hypothermia, electrocution and carbon monoxide poisoning^9^. Animal bites is another potential risk which was seen during the current floods in Pakistan^10^. Disasters could exacerbate non-communicable diseases and might lead to death^11^. This can occur for several reasons. For instance, treatment of existing conditions might be interrupted due to the lack of access to healthcare facilities or unavailability of health services. Shortage of medications, medication non-compliance, poor diet, interruption of glucose monitoring and several other reasons might contribute to the aggravation of chronic diseases^11^. Malnutrition is another major concern during floods. Over 3 million children were reportedly at increased risk of malnutrition in Pakistan as of 31 August 2022^12^. The increasing prices of food commodities might exacerbate this problem^13^. Looking after pregnant women, ensuring safe delivery and access to quality reproductive health services are also great challenges for the healthcare workers amidst such predicaments. The United Nations Population Fund called for maternal support for 650 000 pregnant women living in affected areas^14^. Another great repercussion of floods is increased mental health problems. Depression, anxiety and posttraumatic stress disorder may rise in such events driven by emerging health problems, loosing close people or socioeconomic hardships^15^. Establishing adequate mental healthcare services in the aftermath of floods is therefore of paramount importance.

The outbreak of infectious diseases is arguably among the greatest non-immediate post-flood threats if not the greatest one. Water-borne, vector-borne, vaccine-preventable, respiratory, cutaneous and ocular infections might all arise during and after floods. The lack of access to clean water, damaged sanitation systems, interruption of immunization programs, suspensions of vector-control programs are among the plethora of factors that facilitate the spread of infectious diseases. In this review we will discuss the outbreak of infectious diseases in Pakistan following the floods.

## Water-borne diseases

Floods can pose a serious public health risk in areas where the transmission of infectious diseases is endemic. Floods disrupt sewage systems, water supply, and sanitary infrastructure, which raises the danger of disease transmission. Floods are most likely to impact diseases that rely on either water for transmission between hosts or involve a host/vector in their life cycle, such as water-borne or vector-borne diseases. Drinking contaminated water or coming in contact with it can spread water-borne infections in the aftermath of flooding. Water-borne epidemics have shown an upward trend globally, which is consistent with the rise in flood events. According to existing literature, cholera, cryptosporidiosis, non-specific diarrhoea, rotavirus, typhoid, and paratyphoid fever all increases after floods^16^. In 2010, a cross-sectional survey of 7814 flood victims in Pakistan revealed that 30% of them had digestive system infections, 33% had skin and soft tissue infections, 7% had conjunctivitis, 5% had respiratory tract infections, and 4% had probable malaria^17^. Since most of the flood-affected communities have poor access to clean drinking water, cholera, typhoid, and other water-borne illnesses pose the greatest threat^18^. Of the 33 million Pakistanis hit by the current 2022 floods, it was initially predicted that five million individuals, including children, might become ill in a few weeks’ periods because of the spread of water-borne and vector-borne diseases^19^. Initial reports also showed that 45 districts in Baluchistan, Sindh, Punjab, Khyber Pakhtunkhwa, and the Islamabad Capital Territory have been impacted by an outbreak of acute watery diarrhoea. The most prevalent illnesses among displaced people who have been living in unsanitary conditions for weeks, aside from malaria, are acute diarrhoea, typhoid fever, and skin diseases^6^.

Considering the increasing concern over the spread of water-borne infections among flood victims, the WHO has issued a warning regarding an imminent “second disaster” of disease and death^20^. According to the Sindh Health Department, 2.5 million people have received care at various medical camps throughout the province since 1 July. According to a report by the Sindh Directorate General Health Services, 594 241 people received treatment for skin-related illnesses, followed by diarrhoea (534 800), malaria (10 702), dengue (1401), and other disorders (120 745)^21^.

Floodwater in Pakistan has polluted the water reservoirs and tube wells. Most families who lost their homes are currently residing in tents along the sides of the rivers and canals that are overflowing; the floodwater they may drink is polluted. Toilets are non - operational because they have been demolished or are muddied. Water-borne infections have been documented by Pakistani health officials in places where the catastrophe has impacted tens of thousands. It is necessary to supply safe water and to enhance sanitation conditions to stop a disease outbreak from spreading widely^22^.

An outbreak assessment report raised concerns in September that children living in flood-affected areas have a high risk of contracting leptospirosis^23^. The bacterial zoonotic disease, which is transmitted to humans through contact with infected animals’ urine or with water, food or soil contaminated with the urine, pose a threat to the children expected to be delivered in flood-affected areas. Preventive measures and rodent control strategies should be considered. Infection with hepatitis A & E is also common after floods since the risk of faecal-oral transmission increases as a result of water contamination, damaged sanitation facilities and overcrowding^24^. Securing clean sources of water and sanitary conditions is pivotal for the prevention of the viral hepatitis.

To lessen vulnerability to infectious illnesses, public health measures should be conducted before, during, and after flooding. Individual, household, communal, regional, and national levels must all be involved in interventions. The main public health methods to lower the risk of infectious diseases driven by floods are risk assessments, improved surveillance systems, and particular prevention and control strategies^16,25,26^,

Following flooding, infectious diseases can be prevented by maintaining health services, providing shelter, clean water, good sanitation, a steady supply of food, and, in some situations, mass vaccination drives^16,25,26^, In the days immediately following flooding, providing safe drinking water is a top concern since water and sanitation are essential factors in the transmission of water-borne diseases. Contrary to water source interventions, household interventions are more successful in avoiding diarrhoea^16,27^, The interventions at the household level that were examined include chlorination, filtration, sun disinfection, and combination of flocculation and disinfection. Interventions involving hand washing can cut down on diarrhoea bouts by one-third^28^.

## Vector-borne diseases

Hundreds of thousands of people have been displaced across Pakistan after the floods and are therefore living close to ponds of stagnant water which are formed when floodwater starts receding. Stagnant water provides a nesting spot for mosquitos, thus resulting in a wider spread of mosquito-borne diseases like dengue fever and malaria. The number of dengue cases was surging after the floods, and Sindh province in particular saw a large outbreak of the disease. As of 26 September 2022, 7951 cases were reported in Sindh, 4921 in Punjab, 6625 in Khyber Pakhtunkhwa and 1991 in Islamabad^29^, and the numbers are expected to be worse. Researchers pointed out that 85% of the reported cases in Karachi, the capital of Sindh province, are caused by DENV-1 serotype, whereas DENV-2 accounted for 80% of the cases in 2021, hence the increase in the number of severe cases^30^. The death toll because of this disease reached 31 in Sindh as of 25 September. On 23 September 2022, the Pakistan Meteorological Department expected, based on climate outlook, that a dengue outbreak may occur in October in ten major cities in Pakistan due to the ideal environment for such an outbreak in terms of atmospheric variables^31^. As for malaria, 4000 cases were reported daily in September across Sindh, where 162 395 cases had been reported throughout the year^32^. Forty-four thousand cases were reported in only one week in this province^33^. Away from mosquitoes, both cutaneous and visceral leishmaniasis are endemic in Pakistan and floods might boost the transmission of this disease. A spike in the number of cases was seen earlier in 2022; however, it is not known whether the current floods had led to an increase in the number of cases as data in this regard is lacking, hence caution is warranted at this point.

Although dengue is endemic in Pakistan and malaria is prevalent, numerous factors make this particular battle against these diseases unique and fierce. Contracting fever is common among victims of floods, which might render the identification of potential cases more difficult, though early diagnosis is crucial for effective treatment. In September, there was reportedly a need to secure over 7 million mosquito nets for flood victims^34^. The shortage of mosquito nets and repellents hinders the preventive efforts and makes people more prone to these diseases. The paucity of testing kits and antimalarial medicines is yet another problem to be addressed. These diseases pose a great danger to pregnant women and foetuses, therefore the estimated 650 000 pregnant women living in affected areas should be well taken care of.

The government launched a campaign aimed at raising the public awareness of dengue and took measures to prevent its spread. The community was called upon to support these efforts through getting rid of stagnant water and practicing good hygiene. In Sindh, fumigation campaigns were initiated to prevent the breeding of mosquitoes. Since a vast area of the country was covered by water, it has been suggested that aerial spraying or breeding larvae-eating fish in ponds might be the optimum solution to curb the spread of dengue^35^. Apart from the devastating disaster, the governmental efforts aimed at curbing dengue in Pakistan had not been sufficient even before the floods despite the recurring outbreaks. There is a pressing need to draw lessons from successful stories of outbreak prevention during past floods and improve disaster preparedness plans. Healthcare workers should be trained to deal with such crises, well-equipped laboratories and effective surveillance systems should be established, vector-control programs should be reinforced and the public awareness should be raised^36^.

## Poliomyelitis

In light of Pakistan’s post-flood situation, a strong emphasis on polio becomes necessary in this review paper. This is due to changes in vaccine distribution and the availability of distinct transmission channels. Despite these challenges, polio’s persistence necessitates targeted responses, with rapid vaccination campaigns particularly crucial due to high population density. Treating polio separately emphasizes its global significance, necessitating specialized approaches for the disrupted surveillance system, and compromised healthcare infrastructure post floods. Polio is a highly transmissible virus that the WHO is attempting to eradicate^37^. While the virus is mostly transmitted by the faecal-oral route, other modes of transmission include contaminated consumables, drinks, and, to a lesser extent, airborne droplets expelled through sneezing and coughing^38^.

Poliomyelitis is still endemic in Pakistan and Afghanistan. Both the wild poliovirus type 1 (WPV1) and the circulating vaccine-derived poliovirus type 2 (cVDPV2) affect Pakistan. The health authorities declared on January 27, 2022, that a year has elapsed since the last reported case of polio in the country. It was not long before an outbreak struck in April resulting in 11 cases. As of September 2022, 19 WPV1 cases have been documented in the country, all in Khyber Pakhtunkhwa province; 16 in North Waziristan district, 1 in South Waziristan and 2 in Lakki Marwat district^38^. A 20^th^ case was reported on 30 September in North Wazirstan district^39^.

The Tochi River runs from Afghanistan’s Paktika province, where one case of polio was detected in January, and enters Pakistan’s Khyber Pakhtunkhwa province flowing through all the districts where the 20 cases were detected this year^40^. The river ultimately joins the Indus River which spans the entirety of Pakistan and connects all its main rivers. This connection enables the poliovirus to propagate since it is known to circulate through water channels in the environment. Although all the cases have been detected only in Khyber Pakhtunkhwa province so far, environmental samples from nine major cities in different provinces have already showed viral transmission, therefore the spread of the virus to the populous cities is dreaded^41^.

The supplementary immunization activities (SIAs) targeting polio in particular were launched in 1994 in the country^42^. The national monitoring of acute flaccid paralysis was initiated the same year, but only became operational in 1998. Beginning from 2000, immunization campaigns included house-to-house visits with at least 7 rounds of national vaccination days in response to disease outbreaks or local surveillance data^43^. Since the beginning of Pakistan’s Polio Eradication Program in 1994, the number of polio cases has drastically decreased, from around 20 000 reported cases in the early nineties to only 8 cases in 2018^44^. Polio eradication in Pakistan was on the verge of being accomplished, particularly in 2018, but unforeseen events, such as the COVID-19 pandemic, have got in the way. In addition to the pandemic, there are numerous other challenges to the polio campaign and SIAs. Natural disasters, prejudice against the polio vaccine, war and conflicts, a decline in vaccine providers, ignorance, political and social concerns, the Afghan conflict, and ineffective vaccines are the main obstacles in the way of polio eradication in Pakistan^45^. Sadly, polio teams have been repeatedly attacked and shot dead during immunization campaigns, the last of which was in June 2022^46^. Although vaccination coverage is estimated at 95%, some areas still report low vaccination rates despite the fact that less than 1% of the people refuse to get the polio vaccine nationwide^47^.

Natural disasters pose a substantial challenge towards the drive of the polio immunization campaigns. The third annual nationwide polio campaign began on 22 August in 108 districts of Pakistan, with the goal of providing the vaccine to 43.3 million children^48^. Polio frontline workers had already started their campaign activities in Karachi and South Khyber Pakhtunkhwa earlier on 15 August due to the floods and other hazards of viral transmission. However, campaigns were disrupted in flood-affected areas^49^. In light of the rising number of positive environmental samples and cases, there is an imperative and urgent need to strengthen polio surveillance, stop the circulation in the rivers, reinitiate immunization campaigns, provide access to clean water and sanitary facilities in the ravaged areas.

## Respiratory diseases

Floods displace people from their homes. In a bid to salvage the situation and provide adequate shelter, flood victims are placed in makeshift camps. However, the crowding of these people in these areas exposes them and make them more vulnerable to various health problems of which respiratory infections are not left out^50^. In flood-affected areas, standing water helps bacteria, viruses, and mould breed which could become airborne. Inhalation of these microorganisms put individuals at risk for lung diseases which can easily be spread from person to person^51^. The spread of respiratory diseases occurs through droplets, person-to-person contact, touching infected fomites, and subsequently touching the nose or mouth^52^. A study conducted in Bangladesh in 1988 investigated the cause of illness in 46,740 patients after one of the worst floods in the country’s history and found that respiratory tract infections accounted for 17.4% of total cases and contributed to 13% of all deaths^53^. The 2010 floods in Pakistan saw ~5.6 million patients reporting diseases. Out of this, respiratory infections came second (15.1%) only to skin infections (18.3%) as the leading cause of morbidity in the country^54^.

The 2022 floods in Pakistan led to the outbreak of respiratory tract infections^55^. According to Sindh health department, some 13 989 cases of acute respiratory infections were detected in the province^53^. COVID-19, which has become a global burden in recent years, was rising in Pakistan. This is hardly surprising given the overcrowded camps and non-compliance with social distancing measures, which facilitates the spread of viral respiratory infections^56^. Twenty thousand one hundred sixty-four cases of COVID-19 were reported across the country in a span of 3 months before the floods (1 March–31 May 2022)^57^, while within 3 months during and post the floods (2 June–30 August 2022) 38 331 cases were recorded, representing 90% increase in the number of newly reported cases. Aside from COVID-19, diphtheria, a vaccine-preventable disease, claimed the lives of 10 children in Sindh province and 39 cases have been confirmed and the numbers are suspected to be higher^58^. The lack of experience in dealing with this disease is hampering the management of the cases. In the wake of the Kosi River flood in India in 2008, new cases of tuberculosis were reported^59^. Pakistani health officials should therefore remain vigilant about the emergence of new cases.

As the devastating effects of the floods are expected to increase in the coming months, there is a need to institute safety measures to reduce the spread of respiratory infections in the country. The WHO has made some steps to mitigate infection transmission by diverting mobile medical camps and COVID-19 teams to affected districts and providing kits for collecting clinical samples for early disease detection^60^. The prevention of infectious diseases should be a continuum, starting pre-flood to the post-flood period. This encompass adequate planning, policy development, and continuous education^61^. In areas where there is no pre-disaster surveillance policy, rapid implementation of control measures should be a priority. Surveillance should be done in disaster areas, camps, and health facilities to tackle the emerging disease^62^. Although the implementation of social distancing is difficult in a displaced group of people due to the proximity of living and limited resources^63^, it is essential to ensure the wearing of masks to control the spread of COVID-19 and other respiratory infections^64^.

## Skin and eye infections

Skin and eye infections might occur after coming into contact with contaminated water^24^. Floods might also increase the chance of buildings contamination with mould, increasing thereby the risk of contracting mould-related skin infections. In the aftermath of the 2010 floods in Pakistan, 143 870 skin infections were reported in Sindh^24^. Although these infections are very common after floods, they do not usually provoke epidemics. On 25 September 2022, 1043 skin infections and 165 eye infections were reported in Balochistan within 24 hours^65^.

## Conclusion and recommendations

The thorough analysis provided in this review paper highlights the urgent need for a holistic strategy to address the numerous health issues brought on by the disastrous floods that swept over Pakistan in 2022. The anticipated long-term hazards and the acute health concerns illustrate how crucial precautionary measures and well-coordinated strategies are. Numerous recommendations are made in light of the profound findings the study has revealed.

Firstly, the development of a reliable and responsive catastrophe response structure is crucial. This framework ought to include prompt surveillance and early warning systems for illnesses that are transmitted by water, such as cholera, cryptosporidiosis, severe diarrhoea, rotavirus infection, and enteric fevers. Improved sanitation, hygiene, and water purification techniques ought to be integrated into public health initiatives if we are to prevent and mitigate the spread of these diseases.

Secondly, a greater focus on intensified vector management measures is required to combat illnesses like dengue fever and malaria. Effective vector-control strategies are essential to stop the spread of these illnesses and safeguard public health in Pakistan because of the country’s susceptibility to them. Corporatization of public hospitals in many developing countries represent a fair organizational reform strategy for health system to increase efficiency and satisfaction during the emerging health problems^66,67^

Additionally, it is crucial to prioritize thorough vaccination efforts against diseases like poliomyelitis, COVID-19, diphtheria, and tuberculosis in order to prevent a potential recurrence and boost population immunity. Initiatives to raise disease awareness can also equip communities with the knowledge they need to protect themselves.

Floods increase the likelihood of skin and eye infections, which emphasizes the need to have rapid access to healthcare services and be able to deal with urgent health conditions.

**Figure 1:**
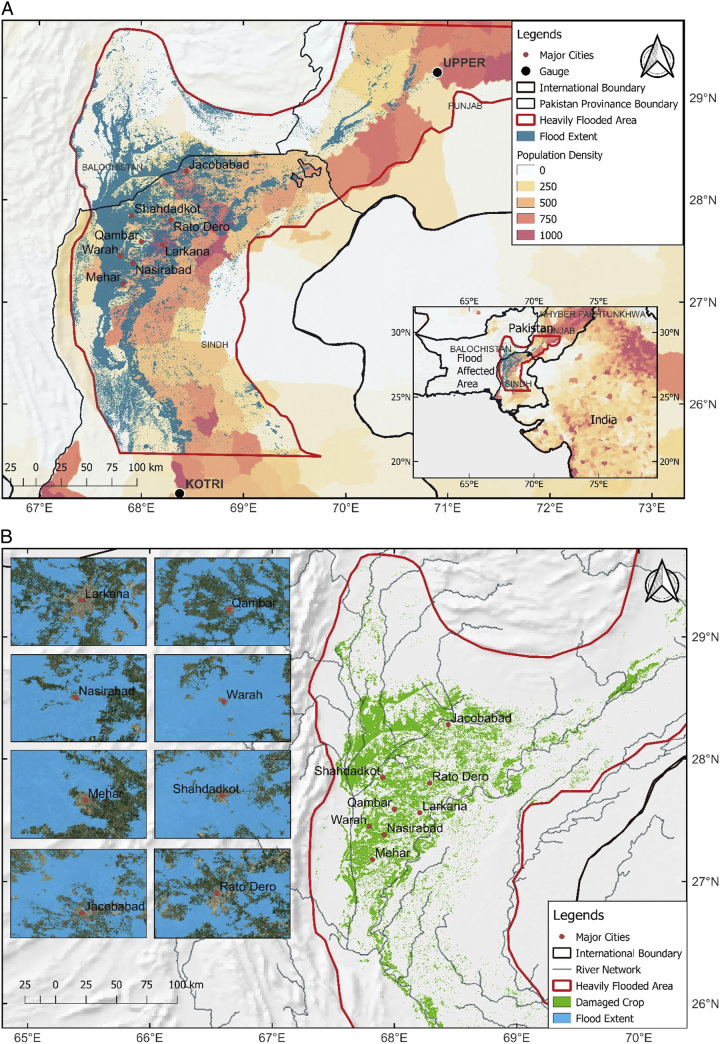
Earth’s Future, Volume: 11, Issue: 3, First published: 13 March 2023, DOI: (10.1029/2022EF003230).

This review highlights the importance of taking concerted, determined action to address the growing health risks that are made worse by climate-related disasters. It highlights how urgent it is for communities, governments, and international organizations to work together to strengthen flood preparedness and response strategies. Additionally, it emphasizes the fundamental necessity of tackling climate change in order to lessen the frequency and severity of such occurrences. The aftermath of the floods in 2022 should serve as a powerful wake-up call for the international community to reassess its commitment to the health and well-being of disaster-prone areas and the general world population (Fig. 1).

## Ethical approval

The present study pertains to a comprehensive literature review on the outbreak of disease following the catastrophic flash floods that occurred in 2022. It is important to note that no patients were directly involved in this study and therefore, the research does not require ethical approval. Rather, the focus is on analyzing and synthesizing the available information from scholarly articles, research papers, and other relevant sources in order to better understand the public health implications of this natural disaster.

## Consent

It is important to note that the present study did not involve the participation of any patients or volunteers. As such, it falls outside the scope of research that typically requires formal ethics committee approval and fully informed written consent from participants.

## Source of funding

This research did not receive any specific grant from funding agencies in the public, commercial, or not-for-profit sectors. Pak Flood Health Crisis 2022: Cholera, diarrhoea, malaria, dengue, polio, COVID-19.

## Author contribution

The study is the result of significant contributions by its authors, with each member playing a critical role in the research process. M.A. led the study by designing the concept and writing the abstract, introduction, and public health crisis sections. A.S., L.T., P.A.K., and S.M.S. contributed by writing the sections on water-borne, vector-borne, respiratory, and poliomyelitis diseases, respectively. Finally, N.T.H. has provided technical review, ensuring the manuscript’s accuracy and readability. S.M.S. led and managed the submission, revised the manuscript, and addressed reviewers’ comments. These interdisciplinary contributions advance our understanding of the disease outbreak post-2022 flash floods.

## Conflicts of interest disclosure

The authors declare that they have no known competing financial interests or personal relationships that could have appeared to influence the work reported in this paper.

## Research registration unique identifying number (UIN)

As this is not a human study(the research did not involve human participant), a Research Registration Unique Identifying Number (UIN) is not required.

## Guarantor

The authors accept full responsibility for the work and the conduct of the study. This includes having access to the data and controlling the decision to publish. This responsibility reflects the authors' commitment to scientific integrity and ensures that the research is conducted with the highest standards of quality and ethical responsibility.

## Data availability statement

The authors confirm the availability of datasets generated or analyzed during the study, either publicly or upon reasonable request, with clear indications in cases where data sharing is not applicable. This commitment to transparency and data sharing is crucial in promoting scientific reproducibility and advancing knowledge.

## Provenance and peer review

If our paper is published, we will include a statement to confirm that it was not commissioned and externally peer-reviewed. This statement is important to clarify that the study was not sponsored or influenced by any external parties and that the research underwent an internal peer-review process.
